# Extracellular Vesicles as Emerging Therapeutic Strategies in Spinal Cord Injury: Ready to Go

**DOI:** 10.3390/biomedicines13051262

**Published:** 2025-05-21

**Authors:** Jiali Jiang, Ziyi Wang, Qinghua Bao, Shenyuan Chen, Wenrong Xu, Jiajia Jiang

**Affiliations:** 1 Aoyang Institute of Cancer, Affiliated Aoyang Hospital of Jiangsu University, 279 Jingang Road, Suzhou 215600, China; lienne_1@163.com (J.J.); ziyi.w.iris@gmail.com (Z.W.); baoqinghua201817@sohu.com (Q.B.); chenshenyuan133238@163.com (S.C.); 2 Zhenjiang Key Laboratory of High Technology Research on sEVs Foundation and Transformation Application, School of Medicine, Jiangsu University, 301 Xuefu Road, Zhenjiang 212013, China

**Keywords:** extracellular vesicle, spinal cord injury, biomaterials, cell-free therapy, bioengineering

## Abstract

Spinal cord injury (SCI) is a prevalent central nervous system disorder that causes significant disability and mortality. Unfortunately, due to the complex pathophysiological mechanisms involved, there remains a critical paucity of effective therapeutic interventions capable of achieving neural tissue regeneration and functional recovery enhancement in SCI patients. The advancements in extracellular vesicles (EVs) as a cell-free therapy for SCI have displayed notable benefits. These include their small size, low immunogenicity, capacity to target specific areas, and ability to cross the blood‒brain barrier (BBB). EVs offer the potential to not only repair tissue damage and stimulate regeneration but also effectively deliver and release them at the site of SCI when combined with diverse biomaterials. This review explores the biological role and importance of EVs in treating SCI, highlighting the combined use of modified EVs with different biomaterials and their potential for future applications. It presents new and hopeful treatment approaches for individuals afflicted with SCI.

## 1. Introduction

Spinal cord injury (SCI) is a severe injury that results in permanent damage to motor dysfunction and sensory perception and causes various complications, such as neuropathic pain and cardiac arrhythmias [1]. Globally, SCI affects more than 20 million people, with different countries reporting an annual incidence of approximately 23.77 cases per million people [2,3]. This condition significantly reduces the quality of life for patients, leading to significant financial and psychological burdens. In clinical practice, the primary therapeutic measures following SCI include prompt surgical spinal decompression, medication, and rehabilitation [4,5]. While recent clinical research has improved the therapeutic outcomes for SCI patients, there are still limitations in fully restoring spinal cord function. Therefore, it is crucial to develop novel therapeutic approaches that promote spinal cord recovery.

Extracellular vesicles (EVs) are a promising and highly recognized new method for repairing tissue damage following cell therapy. EVs are lipid bilayer vesicles secreted by almost all cell types, carrying various biomolecules such as nucleic acids, proteins, and lipids [6]. EVs play a role as “intercellular messengers” in various biological processes such as immune responses, tissue repair, tumorigenesis, and metastasis by transporting these biomolecules [7,8]. The International Society for Extracellular Vesicles (ISEV) recommends dividing EVs into large EVs (>200 nm) and small EVs (<200 nm) based on their size [9]. Large EVs are derived from cell membrane budding, including microvesicles and apoptotic bodies [10]. Small EVs are membrane-bound vesicles released into the extracellular environment after the fusion of multivesicular bodies (MVBs), carrying functional molecules of the parent cells [11,12]. Of note, there is still a lack of recognized separation methods. Large EVs and small EVs cannot be strictly separated and often have cross contamination [9]. EVs released by different types of cells have a vesicle-like shape and encapsulate multiple biomolecules. However, the characteristics of EVs, such as their size, content composition, and surface markers, vary depending on the cell types they originate from [13,14]. This diversity reflects the influence of different cell sources. Therefore, cells from different sources influence the bioactive components and function of EVs [15,16,17].

In the past, the transplantation of stem cells such as mesenchymal stem cells and neural stem cells has been widely used in the treatment of SCI [18,19]. Stem cells can promote the repair of nerve function by secreting neurotrophic factors and replace damaged cells through their multidirectional differentiation ability. However, stem cell transplantation still has some defects such as low survival rate in vivo, immune rejection and tumorigenic risk, which hinder its clinical transformation [20,21]. In recent years, EVs have emerged as crucial players in the initiation, progression, and recovery of central nervous system injury. Studies have shown that EVs are not only easy to store and transport, but also overcome the immunogenicity and tumorigenicity of cell therapy [7]. Specifically, EVs possess the ability to enhance the regenerative capacity of neurons and improve the external microenvironment in the injured area following SCI [22,23,24]. Additionally, in order to achieve sufficient targeting and prolong the half-time of EVs, engineered EVs strategy combined with targeted peptide modification and biomaterials has become a hot topic in SCI [25,26,27]. In view of the complex pathophysiological mechanism of SCI, the multi-targeted combination therapy strategy can avoid the limitations of single therapy and significantly enhance the homing ability, enrichment efficiency, and sustained release of EVs at the injured site. Here, we provide a brief overview of the mechanisms underlying SCI, and summarize the possible therapeutic benefits of EVs derived from various cell types and engineered EVs in treating SCI. Additionally, we will explore the emerging strategies involving the combination of EVs with various biomaterials as a potential treatment approach for SCI.

## 2. Pathophysiology of SCI

**Figure 1:**
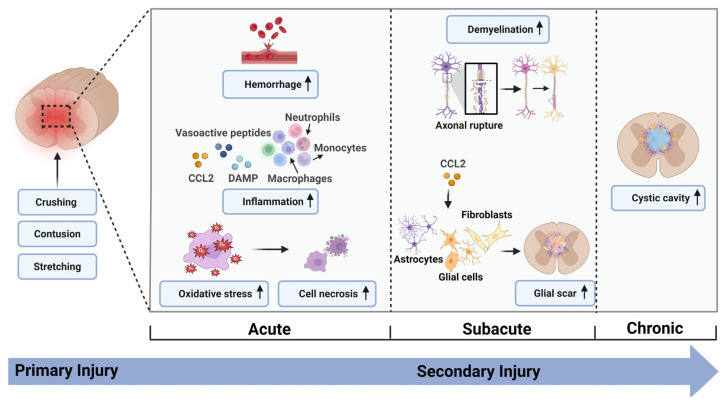
Pathological physiology and fundamental mechanisms of SCI at different stages of the injury. (All figures were created with BioRender.com).

Based on the underlying pathophysiology, SCI can be categorized into primary injury and secondary injury (Figure 1). Primary injury, which is the disruption of the spinal cord’s structural integrity caused by physical factors such as compression, contusion, or stretching, causes irreversible mechanical damage, leading to hemorrhage, disruption of the blood–spinal cord barrier (BSCB) and nerve fiber bundles, and spinal shock [28,29,30]. This initial injury is followed by a series of secondary events, including ischemia, hypoxia, inflammatory response, oxidative stress, neuronal death, demyelination, and scar formation, further exacerbating the extent of injury [30,31]. During the acute phase and subacute phase, disruption of the microvasculature and BSCB results in changes to the local microenvironment at the site of the injury, including infiltration of immune cells (e.g., neutrophils, macrophages, and monocytes) and release of inflammatory factors, vasoactive peptides, and damage-associated molecular patterns (DAMP) [32,33]. These factors further intensify the inflammatory cascade response, for example, chemokine CCL2 promotes the migration of peripheral immune cells, activation of microglia, and neuronal apoptosis [34]. Moreover, ischemia induces prolonged edema and vascular thrombosis, leading to the accumulation of excessive reactive oxygen species (ROS), reactive nitrogen species, and other free radicals at the site of the injury, which creates a hypoxic microenvironment and ultimately triggers neuronal apoptosis [35,36]. Subsequently, when the chronic stage of injury is entered, reactive astrocytes aggregate around the cystic cavitation created by necrosis of the spinal cord’s gray matter to form a glial scar. This barrier-like structure may restrict regenerating axons from reaching the injury site, impair neural axon regeneration and tissue repair, and ultimately result in permanent functional deficits [30,37]. Improving the microenvironment of the injury site, regulating oxidative stress and neuroinflammation, and activating endogenous neuronal and axonal regeneration processes are crucial for treating SCI.

Traditionally, surgical procedures, medication administration (such as neurotrophic drugs, dehydrating agents, and hormones), and the incorporation of traditional Chinese medicine approaches are the main clinical treatments for SCI. Unfortunately, there is currently no successful method for repairing damaged nervous tissue and enhancing functional restoration. Recently, researchers have shown a significant interest in EVs as a novel approach for treating SCI. The autocrine, paracrine, and endocrine mechanisms of EVs play a significant role in cellular communication, enabling EVs to effectively cross the BBB and enter the bloodstream through endocytosis. Several studies have shown that EVs, as important mediators of intercellular communication, contribute to promoting axon regeneration, restoring nerve function, and facilitating tissue repair post injury [38,39,40].

## 3. Industrial Production of EVs

EVs, as a cell-free therapy, have received widespread attention in the fields of tissue damage repair and regenerative medicine [41,42,43]. With the advancement of technology and in-depth research, problems such as low yield and inherent heterogeneity of EVs have emerged. The industrial production of EVs is still limited by cultivation methods and separation technologies. The traditional cultivation of EVs involves the production of cells in cell culture flasks, followed by the collection of culture supernatants and the extraction of EVs. This method has limited cultivation space and requires frequent passage with a large amount of labor, which hinders its clinical application [44]. Although 2D cell culture has a simple operation process, the yield of EVs is low and the labor and economic costs are high [45]. Recently, three-dimensional (3D) cell culture systems such as hollow fiber bioreactors, stirred tank bioreactors, suspended bioreactors, and 3D microbeads have achieved mechanical stimulation on cells to promote the biogenesis of EVs [44,45,46]. Under the same conditions, this method can achieve large-scale production of EVs and reduce the manufacturing costs. Three-dimensional cell culture simulates the in vivo microenvironment under physiological conditions and promotes cells–matrix interactions, thereby increasing the yield and functional activity of EVs [44,47]. Studies have shown that compared with 2D culture, the 3D culture of hucMSCs using vertical wheel bioreactors resulted in a 9.4-fold increase in the number of EVs collected per batch and a greater amount of EVs released per cell [48]. In addition, 3D cell culture enriched EVs with more bioactive proteins and miRNAs related to metabolism, immune regulation, and neural growth [49,50]. However, the high shear rates, materials, and structures of bioreactors can affect the yield and functional activity of EVs. Moreover, the production, function, and targeting of EVs could be significantly improved through gene editing technology or drug stimulation [51,52]. However, it also faces challenges such as technical complexity and safety. The large-scale cultivation of gene-edited cells requires a high cost. The drug-stimulated batch processing mode cannot achieve continuous production, limiting clinical applications.

EVs derived from different cell sources have different characteristics of size, content, and surface markers. The traditional methods for EV separation include ultracentrifugation, size exclusion chromatography, ultrafiltration, and immunoprecipitation [53,54]. Of note, ultracentrifugation is regarded as the “gold standard” for EV separation and is widely used for EV separation and purification in various samples [55,56]. However, this technology has limitations such as complex separation procedures, and poor purity and uniformity of EV separation [53,57]. It is crucial to overcome the limitations of EV isolation technology in order to further explore the molecular basis of targeted delivery of EVs in disease treatment. Recently, microfluidic technology based on multiple microchannel single-cell analysis precisely separated EV subpopulations. Compared with traditional methods, microfluidic technology can reduce sample and reagent losses, and improve separation efficiency and accuracy [58,59]. Asymmetric flow field-flow fractionation (AF4) separates EV subpopulations based on differences in fluid flow properties and fluid dynamics, featuring high reproducibility, specificity, rapidity, convenience, and no labeling [60,61]. Liangsupree et al. isolated sEV subpopulations with diameters of less than 50 nm, 50–80 nm, and 80–120 nm using AF4 technology [62]. AF4 separation technology is beneficial for in-depth research on the biogenesis, molecular mechanisms, and metabolic regulation characteristics of EV subpopulations [7,61]. Therefore, effective large-scale production and separation technologies contribute to the production of EVs at clinical-grade quality. Future research needs to formulate standardized production strategies for EVs, which will help achieve breakthrough progress in the treatment of diseases such as SCI.

## 4. The Emerging Roles of EVs from Various Cell Sources in SCI

**Figure 2:**
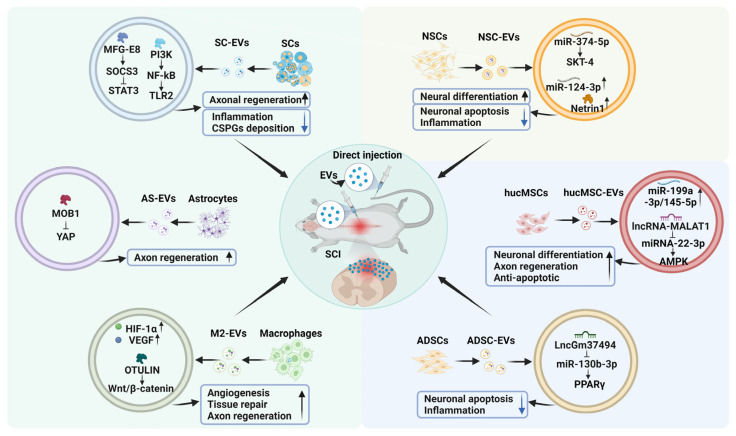
The reparative effects of EVs derived from various cell types on SCI. The diagram depicts different cell sources of EVs, including NSCs, hucMSCs, ADSCs, SCs, astrocytes, and macrophages. EVs, administrated with the tail vein injection or lesions injection, exhibit varying levels of efficacy in generating new neurons to restore neural pathways, promoting angiogenesis, axon regeneration, and tissue repair, preventing apoptosis, reducing inflammation, and excessive growth of glial cells.

Cell therapy has been shown to be a promising therapeutic target for treating refractory diseases. Controversially, its clinical application is limited by immune reactions and high costs associated with storing and transporting reagents [21,63]. To address these challenges, researchers have turned to cell-free therapies, specifically exploring EVs from various cell sources as a safer alternative. EVs from various cell sources, such as neural stem cells (NSCs) and mesenchymal stem cells (MSCs), such as human umbilical cord MSCs (hucMSCs) and adipose-derived stem cells (ADSCs), have demonstrated immunomodulation, anti-inflammation, tissue repair, and regeneration properties [16,64,65]. In the treatment of SCI, EVs offer distinct advantages over cell therapy [17,66,67]. In this discussion, we mainly examine the importance of EVs derived from different cell sources, such as NSCs and MSCs, as well as Schwann cells (SCs), astrocytes, and macrophages (Figure 2).

### 4.1. NSC-Derived EVs

NSC-derived extracellular vesicles (NSC-EVs) have multifaceted biological capacities, including regulating microglial polarization, inducing neural proliferation, and maintaining cellular homeostasis [16,68,69]. They offer a promising therapeutic avenue for nerve repair and regeneration. For instance, NSC-sEVs are enriched with Netrin1, which promotes neural differentiation functionality by upregulating the expression of transcription factors Hand2 and Phox2b through the Hand2/Phox2b axis [70]. miR-374-5p highly expressed in NSC-sEVs can promote autophagy and inhibit neuronal apoptosis through the regulation of the target gene SKT-4, alleviating spinal cord injury [71]. miR-124-3p abundance in NSC-sEVs can mediate the PI3K/AKT/NF-κB signaling pathway, which leads to the suppression of pro-inflammatory microglia and astrocyte activation, thus facilitating the recovery of motor function after SCI [72]. Altogether, NSC-sEVs exhibit anti-inflammatory properties, inhibit neuronal apoptosis, and establish a conducive microenvironment for neuronal differentiation and tissue regeneration.

### 4.2. MSC-Derived EVs

MSCs have aided significant advancement in tissue engineering and cell therapy due to their characteristics of multidirectional differentiation and paracrine signaling. At the onset of acute injury, factors such as IL-4, IL-13, and CCL2 secreted by MSCs regulate macrophage polarization, thereby reducing the extent of secondary injury and promoting functional recovery after SCI [73,74]. Compared with stem cells from other sources, hucMSCs have great potential in immunomodulation and regeneration [75,76,77]. HucMSC-derived sEVs (hucMSC-sEVs) have unique advantages, such as easy access, high efficiency of amplification and culture in vitro, and low immunogenicity [41,78]. miR-199a-3p/145-5p abundance in hucMSC-sEVs affects the ubiquitination of the TrkA by NGF/TrkA pathway, thereby inhibiting the detrimental effects of LPS on neuronal differentiation and promoting axon regeneration [64]. Interestingly, under the condition of microelectric fields, hucMSC-sEVs enriched with lncRNA-MALAT1 promoted neural tissue repair and motor function recovery through the miRNA-22-3p/SIRT1/AMPK axis [79].

sEVs derived from ADSC (ADSC-sEVs) contain neurotrophic, immunomodulatory, and angiogenic factors. These vesicles promote cell proliferation, alleviate scar tissue formation, prevent neuronal cell death, and decrease inflammation through paracrine signaling [80,81]. Moreover, under hypoxic conditions ADSCs secrete more sEVs (ADSC-Hyp-sEVs). LncGm37494 enriched in ADSC-Hyp-sEVs can target and inhibit miR-130b-3p and upregulate PPARγ expression, thereby promoting microglial polarization and alleviating the inflammatory microenvironment [65]. Collectively, the strengths of ADSC-sEVs in promoting cell proliferation and inhibiting neuronal apoptosis and inflammatory responses provide valuable insights for the treatment of SCI [82,83].

### 4.3. EVs Derived from Other Cells

SCI involves complex pathophysiological mechanisms, such as immune regulation involving macrophages and neurotoxicity mediated by A1-type astrocytes. Mounting studies suggest that EVs derived from different cell types, including SCs, astrocytes, and macrophages, are valuable in promoting SCI functional recovery.

SCs, as glial cells, facilitate axon regeneration and myelin formation and maintain the structural integrity of axons [84,85]. Schwann cell-derived sEVs (SC-sEVs) increase mitochondrial autophagy and reduce necrotic apoptosis through the AMPK signaling pathway [86]. SC-sEVs also promote TLR2 expression in astrocytes and downregulate the deposition of chondroitin sulfate proteoglycans (CSPGs) by activating the NF-κB/PI3K axis, promoting nerve functional recovery after SCI [85]. MFG-E8 is highly enriched in SC-sEVs, which can promote the polarization of M2 macrophages and microglia, and attenuate inflammatory responses by activating SOCS3 and inhibiting STAT3 phosphorylation [66].

In the later stage of SCI, pericytes and fibroblasts that accumulate at the site of injury are the “main force” of a glial scar. The presence of glial scarring restricts axonal growth and extension, which is ultimately detrimental to nerve functional recovery. Astrocytes exhibit significant advantages in neuroprotection [87]. For example, astrocyte-derived extracellular vesicles (AS-EVs) can increase the expression of MOB1 and the phosphorylation of YAP by activating the Hippo pathway, thus promoting neuronal elongation and recovery [88]. Additionally, AS-sEVs can reduce fibrosis in injured tissues and promote tissue repair and motor functional recovery [17,89].

Due to the destruction of the spinal cord vascular network, ischemia and cell death after SCI are challenging and lead to spinal cord dysfunction. Many studies have reported the role of growth factors in neurological diseases. M2 macrophages are conducive to new blood vessel formation and facilitating nerve development following SCI [90,91]. It is reported that M2 macrophage-derived EVs enriched with glial cell line-derived neurotrophic factor (GDNF) collected by transfection can significantly promote the outgrowth of axons and dendrites [92]. Additionally, M2-sEVs can promote the migration and proliferation of cerebral endothelial cells by upregulating proangiogenic factors HIF-1α and VEGF, thus facilitating vascular repair and nerve regeneration after SCI [67]. The ubiquitin thioesterase (OTULIN), which is highly enriched in M2-sEVs, mediates the activation of the Wnt/β-catenin axis and induces angiogenesis and tissue regeneration at the site of injury [90]. Interestingly, research has shown that neurotrophic factor basic fibroblast growth factor (bFGF) increases the release of neuronal EVs and the enrichment of various proteins by regulating mechanisms related to vesicle fusion, such as calcium and VAMP3 [93].

In addition to the EVs from different cell sources mentioned above, EVs secreted by Treg cells, M2 microglia, endothelial cells, etc., also play beneficial roles in inhibiting neuroinflammation, enhancing axonal regeneration, and promoting neurological recovery [94,95,96]. However, the clinical application of EVs still faces multiple limitations. For example, systemic administration such as the tail vein injection of EVs accelerates the capture and clearance of EVs by the mononuclear phagocytic cell system. A small amount of EVs can cross the blood–brain barrier and reach the site of spinal cord injury [97]. In addition, although EVs have lower immunogenicity than cell therapy, their surfaces may still carry major histocompatibility complex (MHC) molecules from the donor cells, causing host immune rejection. The safety of long-term multiple administration still needs to be verified [98]. Existing SCI research is mostly based on rodent models, whose damage mechanisms and repair abilities differ from those of humans, which may overestimate the therapeutic efficacy of EVs. The resolutions of these obstacles are crucial for establishing EVs as a dependable option for clinical SCI treatment.

## 5. Application of Engineered EVs in SCI Treatment

For a long time, we have been perplexed by the clinical challenges of SCI and the need to improve patient prognosis and quality of life. In view of their advantages of low cytotoxicity, low immunogenicity, and high biocompatibility, EVs have received extensive attention from researchers as important carriers for intercellular communication [99,100]. Recently, engineered EVs modified with peptides or loaded with contents have shown advantages in targeted delivery and in vivo recycling, providing an economical, convenient, and effective treatment for SCI.

Engineered EVs with surface-immobilized specific peptide segments can target and bind to specific cell receptors at the injury site, thereby enhancing EVs’ homing ability [27,101]. Exo-pep-11 was constructed by chemically coupling the EphA4 receptor targeting peptide with ADSC-sEVs, which could specifically bind to NSCs and stimulate its proliferation and differentiation [102]. For instance, it has been discovered that the intranasal administration of RGD (Arg-Gly-Asp) peptide-modified EVs could target endothelial cells overexpressing integrin α_v_β_3_ at ischemic regions of SCI, significantly increasing the specific accumulation of EVs at the lesion site and contributing to the stability of the BSCB [27].

Exogenous drugs, biomolecules, and cytokines can be embedded into EVs through techniques such as electroporation or ultrasound [103]. The CAQK peptide can specifically bind to reactive astrocytes with a high expression of chondroitin sulfate proteoglycans (CSPG) in the lesion area. Based on the CAQK peptide chemical modification of induced neural stem cell (iNSC)-derived EVs, C-EVs-siRNA was constructed by electroporation loaded with CCL2-siRNA. C-EVs-siRNA promoted the synergistic effect of CCL2-siRNA and EVs to target and inhibit inflammation and neuronal apoptosis after SCI [104]. Moreover, EV membranes can protect drugs from enzymatic degradation and escape immune system clearance, thereby improving drug delivery efficiency. FTY720-NSC-sEVs, engineered NSC-sEVs loaded with FTY720, can effectively avoid the serious adverse reactions caused by the systemic administration of FTY720, reduce the expression of AQP-4 at the injury site, and upregulate claudin-5 to synergistically alleviate spinal cord edema [27]. Furthermore, FTY720-NSC-sEVs can mediate the downregulation of PTEN to activate the PI3K/AKT signaling pathway, thereby mitigating the effects of neuronal apoptosis and improving functional recovery after SCI. An engineered EV with neuroprotective and anti-inflammatory abilities, Cur@EVs-cl-NGF, was combined with a nerve growth factor (NGF) and curcumin (Cur) based on M2-sEVs. NGF can effectively enhance the ability of EVs to promote axon extension and inhibit neuronal apoptosis [105]. When Cur@EVs-cl-NGF is transported to the site of the injury, the localized high expression of MMP9 in SCI can efficiently dissociate NGF. Cur@EVs-cl-NGF can effectively solve the problems of low expression of endogenous NGF, short half-life, and low uptake of Cur. It has shown great advantages in relieving inflammation, neuroprotection, and biosafety.

Engineered EVs can effectively utilize drugs with optimal doses and avoid systemic toxicity. However, engineered EVs still have off-target risk. RGD peptides can bind to normal vascular endothelial cells, causing cross reactivity between targeted peptide modified EVs and non-specific tissues [27]. Moreover, EVs loaded with drugs can promote EVs inactivation in addition to reducing cycle stability. Engineered EVs need to improve targeting accuracy, preparation standardization, and preservation stability to facilitate clinical translation.

## 6. EVs Combined with Biomaterials for SCI

**Figure 3:**
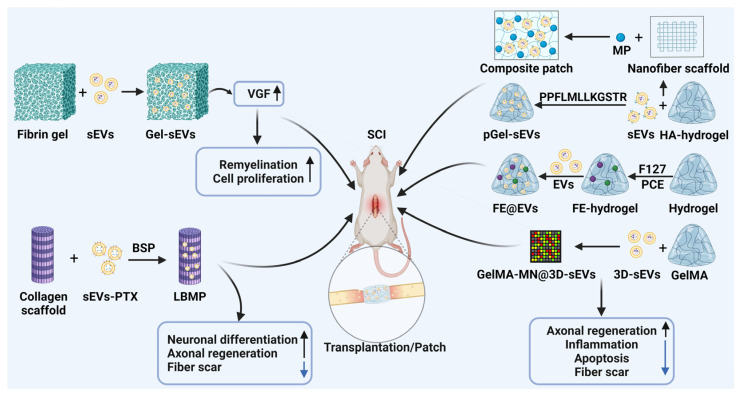
Schematic illustration of EVs binded with biomaterials for SCI. Synthesized composite materials, containing EVs with fibrin gels, collagen scaffolds, and bioactive hydrogels (which are administrated through transplantation or patch), inhibit apoptosis, reduce inflammation, decrease ROS, and promote angiogenesis and axonal regeneration.

Emerging evidence indicates that a local injection of EVs for SCI can be rapidly cleared and metabolized by the human circulatory system. This prevents EVs from remaining at the injured site and releasing continuously, thereby significantly restricting their potential in facilitating the functional recovery of SCI [106,107]. Of note, the implantation of pre-formed biomaterials in situ offers a promising solution for SCI [108]. Injectable biomaterials as targeted delivery carriers can enhance the therapeutic effects of EVs by effectively retaining them at the injury site and facilitating sustained release [109]. Interestingly, significant headway has been made in exploring the application of biomaterials in conjunction with EVs for the treatment of SCI (Figure 3 and Table 1).

Given the potent value of this combination strategy in improving the therapeutic outcomes of EV-based treatments for SCI, various clinical trials evaluating the efficacy of biomaterials in the interventional therapy of SCI are emerging. Table 2 summarizes the current data on biomaterials used in interventional treatments for SCI from domestic and international clinical trials. Currently, most clinical trials involving the use of MSCs combined with biomaterials are still in their early stages. Moreover, clinical studies on EVs combined with biomaterials have not yet been initiated. However, we anticipate that as more research data emerge, the combination of EVs with different biomaterials will become widely adopted for SCI treatment and eventually transition to clinical practice.

### 6.1. EVs Combined with Fibrin Gels

Fibrin, as an FDA-approved biomaterial, has received wide attention. It has high biosafety, pro-wound healing, and degradability properties, which may make it a good scaffold for the repairment of SCI [111,118,119]. Interestingly, the degradation products of fibrin gels have also been shown to be biologically active and can promote cell proliferation, adhesion, and collagen synthesis [120].

Fibrin gels have been reported to enhance the differentiation and proliferation of NSCs and MSCs [108,121,122]. Fibrin gels can provide a matrix for the targeted delivery of sEVs and promote nerve growth, thereby significantly enhancing the role of sEVs in alleviating the microenvironment at the lesion site [110]. Additionally, nerve growth factor inducible (VGF) enriched in MSC-sEVs plays a key role in promoting remyelination and oligodendrogenesis. MSC-sEVs encapsulated in fibrin gel (Gel-sEVs) significantly promoted the expression levels of neural nucleus (NeuN), neural class III β-Tubulin (Tuj1), and doublecortin (Dcx), thereby effectively promoting neurogenesis and the recovery of motor function in SCI [111]. However, fibrin hydrogels, as a biocarrier for sEV loading, have not been explored for their effective retention of sEVs and slow release to the injured site. The degradation rate and mechanical properties of fibrin gel still need to be further improved [110,123].

### 6.2. EVs Combined with Collagen Scaffolds

The main element of the extracellular matrix is collagen, which is biodegradable. Collagen scaffolds possess three-dimensional porous characteristics and are superior to other biomaterials in promoting cell adhesion and growth properties [112,124,125]. The structure of collagen scaffolds benefits material exchange and provides a conducive environment for cell growth and differentiation.

A novel bio-specificity peptide (BSP) can be specifically bound to MSC-sEVs encapsulated with paclitaxel (PTX) and a linearly ordered collagen scaffold, thereby constructing a multifunctional collagen scaffold (LBMP). Compared with traditional binding methods, such as physical adsorption and chemical coupling, BSP, as a versatile linker molecule, is able to effectively preserve the integrity of the sEV membrane and its surface collagen [113]. After 7 days of LBMP implantation, the slow releasing rate of the drug reached 70%. After 60 days, LBMP gradually degraded with tissue regeneration at the lesion site. In the LBMP, MSC-sEVs promoted the migration of endogenous NSCs, while collagen scaffolds could effectively retain NSCs. LBMP has significant advantages in synergistically inducing neuronal differentiation, axonal growth, and inhibiting fiber scar deposition. Remarkably, collagen scaffolds still have problems such as poor mechanical properties [126]. Exogenous peptides in collagen scaffolds are difficult to remove and may cause biosafety concerns [112]. The current studies on the immunogenicity of collagen are also insufficiently in depth and need to be further explored to facilitate the advancement of collagen scaffolds in the clinical treatment of SCI [127].

### 6.3. EVs Combined with Bioactive Hydrogels

Hydrogels, as mimics of the natural extracellular matrix (ECM), possess a nano arrangement with mucoadhesive, elastic, and neuroinductive properties [107,128,129]. By injecting hydrogels with a sustained release of EVs, they can effectively fill the gap at the site of SCI and deliver EVs specifically to the injury site for therapeutic action [115,130].

#### 6.3.1. EVs Combined with Hyaluronic Acid Hydrogels

Hyaluronic acid (HA) regulates a range of physiological processes including inflammation, angiogenesis, and wound healing. HA is present in almost all human tissues and body fluids and has high biocompatibility. However, HA is non-adhesive by itself and needs to be combined with biomaterials such as hydrogels and nanoparticles to expand its application [131,132]. In recent years, researchers have designed novel adhesive peptide-modified HA hydrogels, which optimize the SCI therapeutics involved in existing studies.

Based on the high adhesion of integrins on sEV membranes in combination with laminin, sEVs were implanted into HA hydrogel scaffolds (pGel-sEVs) modified with the laminin-derived peptide PPFLMLLKGSTR [117]. The adhesion rate of sEVs was much higher than that of the hydrogel in the peptide-modified pGel group. The sustained release of sEVs in pGel could be maintained for 11 days, with a cumulative release surpassing 90%. In contrast to equivalent intravenous sEVs via the tail vein, pGel-embedded sEVs demonstrated a diffusion pattern radiating from the lesion center to the periphery, displaying a strong correlation with the lesion site. Recently, a composite patch has been developed for the sequential release of SCs-sEVs and methylprednisolone (MP) at different pathological stages of SCI [35]. There is no significant difference in the properties of sEVs released by the composite patch compared with native sEVs. This composite patch can induce M2 macrophage polarization through the TLR4/NF-κB and MAPK signaling axis and modulate the Akt/mTOR signaling axis to inhibit neuronal apoptosis, leading to an improved microenvironment following SCI. Compared with traditional invasive treatment strategies such as local injection, scaffold implantation, and microneedle array patch, this transdermal drug delivery patch can reduce secondary losses and achieve a painless treatment of SCI [102,113,133]. Collectively, the hydrogel serves as a natural carrier for the sustained release and retention of sEVs, effectively filling irregular cavities at the site of the SCI and preventing secondary damage [116]. Notably, the binding and release of integrins and adhesion proteins in sEVs can be unstable. The release of sEVs following pGel-sEV implantation is influenced by the local microenvironment at the injury site, highlighting the significance of stable and continuous sEV release for effective SCI treatment [117].

#### 6.3.2. EVs Combined with FE Hydrogels

In view of the disadvantages of hydrogels that affect neuronal development, such as poor mechanical properties and low resistance [134,135], it is imperative to enhance the performance of hydrogel. Wang et al. [115] prepared FE hydrogels consisting of F127 and PCE polymers, which are injectable, biocompatible, adhesive, and self-repairing. The MSCs-derived EVs (FE@EVs) loaded into the FE hydrogels by electrostatic interactions showed good, stable release compared with free MSCs-EVs, and approximately 90% of the EVs could be released from the FE hydrogels to the injury site for 56 days. In addition, FE@EVs significantly alleviate the injured microenvironment via activating MAP-2 and neurofilaments, inhibiting fiber scar formation and promoting axonal regeneration.

#### 6.3.3. EVs Combined with Gelatin Methacrylate Hydrogels

Gelatin methacryloyl hydrogels (GelMA) consist of methacrylic anhydride and gelatin, which have good biocompatibility, thermal stability, and adaptability to various chemical modifications [136,137]. GelMA hydrogel was mixed with 3D-cultured MSCs-derived sEVs (3D-sEVs) to make microarray patches with nanostructures (GelMA-MN@3D-sEVs) [114]. According to quantitative proteomics analysis, 3D-sEVs are enriched with more proteins and miRNAs in the modulation of a local microenvironment and neural repair compared with 2D-sEVs. Additionally, 3D-sEVs showed an enrichment of the phospholipase D signaling pathway associated with axon growth and synapse development, as well as the Fc-epsilon-RI and mTOR signaling pathways associated with the regulation of neuroinflammation. Although specific mechanisms have not been elucidated, the combination of GelMA-MN and 3D-sEVs holds significant promise for the guidance of SCI clinical therapy.

The combination of EVs with different biomaterials as a new strategy in regenerative medicine has been widely used in studies on tissue regeneration and functional recovery. In addition, synthetic polymer scaffolds serve as another type of biomaterial that can repair or fill SCI-diseased tissues [138,139]. The synthetic polymers reported in existing research mainly include polyethylene glycol (PEG), polylactic acid (PLA), polyvinyl alcohol (PVA), and so on. Compared with natural polymers, synthetic polymer scaffolds have stable molecular structures, porosities, and mechanical properties [140,141,142]. Furthermore, synthetic polymers can be designed as biomaterials with corresponding biological properties according to different needs. Nevertheless, many biosafety issues with synthetic polymers limit their clinical applications. For example, the degradation products of synthetic polymers can cause local inflammatory reactions and aggravate tissue damage conditions [141,143].

Although the combined application of EVs and biomaterials has demonstrated significant advantages, its clinical application still faces many challenges. Loading EVs through ultrasound or chemical modification may disrupt the membrane integrity of EVs and induce immunogenicity [144,145]. Although the combined application of EVs with biomaterials such as hydrogels can prolong the release time, it is difficult to change accordingly in line with the different requirements of SCI acute and chronic phases [146,147]. In addition, the porosity and mechanical strength of the biomaterials need to match the physical characteristics of spinal cord tissue [146]. Otherwise, they may compress the lesion site or fail to effectively support axonal regeneration. In future studies, methods for overcoming the limitations of biomaterial applications are vital to the advancement of therapeutic strategies for SCI.

## 7. Conclusions and Outlook

SCI is a complicated process that prevents tissue function recovery after injury, greatly impacting the quality of life of patients. EVs, as good alternative materials capable of overcoming the inherent limitations of cell therapy, have become a focal point in SCI treatment research and have entered the clinical trial stage [78]. A series of studies have demonstrated the vast potential of EVs from various cell sources for tissue injury repair and neural regeneration [148,149]. As a potential cell-free therapy, EVs offer a safe and effective treatment strategy for SCI patients. Currently, the core factor of the slow clinical conversion process is the lack of international unified production and quality control standards for EVs, including separation and purification technology, large-scale production, and storage [150]. Notably, EVs-mediated cell-free therapies still have many obstacles to overcome. Current interdisciplinary technology integration is not yet mature. The non-specific accumulation of engineered EVs in the liver, spleen, and other organs may cause inflammatory reactions and damage normal tissues [151,152]. The limitations of EVs combined with biomaterials in the treatment of SCI need to be gradually overcome through in-depth research. A successful clinical translation will depend on the simultaneous optimization of precise delivery systems, in-depth mechanism analysis, and standardized production [113,120]. In the future, we must develop standardized separation and characterization techniques for EVs and establish production processes that comply with standardized operating procedures. We must also construct systematic toxicology evaluation standards and develop intelligent biomaterials through technological innovation [153]. We believe that with further research, these problems can be resolved.

In conclusion, previous studies have demonstrated the efficacy of EVs and their combined applications in promoting tissue regeneration and functional recovery following SCI. Nevertheless, future studies should aim to provide adequate evidence regarding the safety and reliability of these strategies in clinical settings, ultimately offering a promising treatment strategy for SCI with potential clinical applications.

## Figures and Tables

**Table 1:** Biomaterials for promoting functional recovery of SCI.

Material	Method of Modification	SCI Model	Outcome	Reference
Fibrin gel	Human umbilical cord MSC-sEV and in situ gelate-encapsulated fibrin glue	Spinal cord injury (T9–T11)	Alleviate inflammatory and oxidative microenvironment Induce effective nerve tissue repair and functional recovery	[110]
Fibrin gel	Encapsulation of sEV	Spinal cord injury (T8–T9)	Enhance neurogenesis Remyelination	[111]
Collagen-I (Col-I) scaffold	Immobilization of CBD peptide fused in Lamp2b on the sEV surface	Spinal cord injury (T10)	Inhibit pro-apoptotic genes Provide enough space for cell growth or migration	[112]
Collagen	BSP specific binding to MSC-sEVs	Spinal cord injury (T8)	Enhance neural regeneration Reduce scar deposition	[113]
Gelatin methacryloyl hydrogel	3D-sEV hybrid culture	Spinal cord injury (T9–T11)	Induce inflammation and glial scar	[114]
F127-polycitrate-polyethyleneimine hydrogel (FE)	Encapsulation of EV	Spinal cord injury (T10)	Suppress fibrotic scar formation Promote remyelination and axonal regeneration	[115]
Hyaluronic acid-hydrogel	Encapsulation of MSC-derived sEV, DBM, PDRN, et al.	Spinal cord injury (T10)	Increase regenerative capacity Reduce pro-inflammatory responses and restore BSCB disrupted by SCI Enhance remyelination	[107]
Hyaluronic acid-hydrogel	Encapsulation of hypo-sEV	Spinal cord injury (T9–T10)	Reduce pro-inflammatory responses Increase angiogenesis capacity and nerve regeneration	[116]
Nanofiber scaffold and hyaluronic acid hydrogel composite patch	Loaded with MP and SCs-sEVs	Spinal cord injury (T10)	Reduce neuronal apoptosis Inhibit inflammatory reaction	[35]
Peptide-modified adhesive hydrogel (pGel)	Encapsulation of hMSC-derived sEV	Spinal cord injury (T9–T10)	Mitigate inflammation and oxidation Improve nerve recovery and urinary tissue preservation	[117]

**Table 2:** Clinical trials investigating the use of diverse biomaterials for SCI treatment.

NCT Number	Study Type	Study Phase	Recruiting Status	Biomaterials	SCI Model	Ages	Enrollment
ChiCTR2100043838	Interventional	0	Recruiting	Nerve regeneration collagen scaffold	Spinal cord injury (C6–T12)	18 years to 60 years	30
ChiCTR-INR-17012152	Interventional	New treatment measure clinical study	Active, not recruiting	Functional scaffold	Complete spinal cord injury (T1–T11)	18 years to 60 years	6
NCT03762655	Interventional	Not applicable	Active, not recruiting	Neuro-spinal scaffold	Complete spinal cord injury (T2–T12)	16 years to 70 years	20
NCT02352077	Interventional	Phase 1	Unknown status	Neural regeneration collagen scaffold	Complete spinal cord injury (C5–T12)	18 years to 65 years	30
NCT03966794	Interventional	Phase 2	Unknown status	Functional neural regeneration scaffold	Complete spinal cord injury (C4–T12/L1)	18 years to 60 years	9
NCT03933072	Interventional	Phase 2	Unknown status	Collagen scaffold	Complete spinal cord injury (C5–T10)	16 years to 65 years	2
NCT05967325	Interventional	Not applicable	Recruiting	Functional self-assembling peptide nanofiber hydrogels	Complete spinal cord injury (T1–T12)	18 years to 60 years	15
NCT04132596	Interventional	Not applicable	Completed	Transcutaneous hydrogel electrodes	Spinal cord injury (C4–T12)	18 years and older	12

## Data Availability

Not applicable to this article, as no new data were created or analyzed in this study.

## Abbreviations

SCI	spinal cord injury
EVs	extracellular vesicles
MVBs	multivesicular bodies
BSCB	blood–spinal cord barrier
DAMP	damage-associated molecular patterns
ROS	reactive oxygen species
BBB	blood‒brain barrier
NSCs	neural stem cells
MSCs	mesenchymal stem cells
hucMSCs	human umbilical cord MSCs
ADSCs	adipose-derived stem cells
SCs	Schwann cells
CNS	central nervous system
lncRNA	long non-coding RNA
Hyp-sEVs	sEVs under hypoxic conditions
AS-sEVs	astrocyte-derived small extracellular vesicles
PKC	protein kinase C
M2-sEVs	M2 macrophage-derived small extracellular vesicles
NGF	nerve growth factor
MMP9	metalloproteinase 9
FE	F127-polycitrate-polyethyleneimine hydrogel
PTX	paclitaxel
ECM	extracellular matrix
HA	hyaluronic acid
3D	three-dimensional
MP	methylprednisolone
PEG	polyethylene glycol
PLA	polylactic acid
PVA	polyvinyl alcohol
